# A Bilateral Acetabular Physeal Fracture Treated with External Fixation in an Immature Cat

**DOI:** 10.3390/ani14030379

**Published:** 2024-01-25

**Authors:** Jose Antonio Flores, Gian Luca Rovesti, Jesus Rodriguez-Quiros

**Affiliations:** 1 Hospital Veterinario IVC Evidensia Prïvet, Calle Duero 37, Villaviciosa de Odón, 28670 Madrid, Spain; 2 Clinica Veterinaria M. E. Miller, Via della Costituzione 10, 42025 Cavriago, Italy; glrovesti@gmail.com; 3 Departamento de Medicina y Cirugía Animal, Facultad de Veterinaria, Universidad Complutense de Madrid, Avda. Puerta de Hierro s/n, 28040 Madrid, Spain; jrquiros@ucm.es

**Keywords:** cat, acetabular, external fixation, fracture, immature cat

## Abstract

**Simple Summary:**

This study intended to show the use of external fixation to resolve bilateral acetabular physeal fractures in an immature cat, obtaining a good outcome with a low rate of complications. A great part of this success was because external fixation allows fractures to be stabilised through a minimally invasive procedure. In addition to its relevance as a fixation method, it was possible to show that external fixation provided comfort and a high level of tolerance for the patient during the whole healing period.

**Abstract:**

This study aimed to assess the outcome of a bilateral acetabular physeal fracture treated with external fixation in an immature cat, a surgical technique not usually employed in immature patients. The fixator took 40 days, and it was removed after radiographic bone healing was achieved. No significant complications related to the technique were identified, and the outcome was classified as good based on the functional assessment and pain scales employed. The use of external fixation for stabilising acetabular fractures in immature cats should be considered a viable technical option, especially for minimally invasive stabilisation.

## 1. Introduction

Pelvic fractures in cats account for 22–32% of all skeletal system fractures [1,2]. Among these, acetabular fractures constitute 14–43% of the total [3,4]. Despite this, there is a lack of information related to non-traumatic “acetabular physeal fractures” (APFs). The acetabular physis closes between 20 and 24 weeks of age [1], and it is unknown whether APFs share an etiological link with “Feline Epiphyseal Dysplasia Syndrome”, where spontaneous fractures of the femoral head are described [5,6,7,8] in male patients, typically early-neutered, young, obese [8], and/or hypothyroid [9]. There are no data in the literature supporting a similar theory for the origin of APF.

Conservative treatment of acetabular fractures has traditionally been considered a therapeutic option in immature patients [10]. Non-surgical options, though, are not the best choice for the treatment of this type of fracture [1,10], as the most common complication is the development of osteoarthritis [11]. Piana et al. suggest that displacements of more than 3 mm in fracture reduction are associated with a higher risk of osteoarthritis in the affected joint [12]. However, other authors argue that this decision is more of a biomechanical matter, where factors such as the patient’s level of activity and body weight are important factors for deciding between conservative or surgical treatment [13]. Other complications observed in displaced, non-reduced acetabular fractures include narrowing of the pelvic canal with subsequent constipation, neurological deficits, and pain [4].

Different techniques are described for the treatment of acetabular fractures, with the most commonly used being small fragment plates or locking plates [4,12,13,14,15,16,17] and the combination of screws with polymethyl methacrylate or cerclage wire [1,4,18,19,20]. In cases where surgical repair is not feasible, salvage procedures such as femoral head and neck ostectomy can provide pain relief and preserve limb function [21]. The use of external fixation (EF) as the sole stabilisation method in the treatment of APFs in immature cats has not been described in the literature to our knowledge. While this technique has been described as a complementary method in a single case in dogs [22], it has been widely employed in paediatric patients with traumatic acetabular fractures in human medicine [23,24].

The potential benefits of EF are that it is a minimally invasive approach, reducing the damage to the vascular supply of tissues, avoiding a delay in bone healing [25,26], and providing less postoperative pain [27]. However, the technique requires a good understanding of the principles of its application [25] to avoid complications. The most frequent complications associated with this technique include implant loosening and delayed bone healing [28,29].

This paper describes the surgical technique and the outcome of a case of a bilateral non-traumatic APF in a 5-month-old cat.

## 2. Materials and Methods

This study involves a 20-week-old, 950 grs cat admitted to Prïvet Veterinary Hospital (Villaviciosa de Odón, Madrid, Spain). The patient presented with difficulty standing and walking, exhibiting evident signs of pain during the physical examination. The report includes details about the cause of the fracture, the surgical procedure, the radiographic images, and the evaluation of lameness both before and after the intervention. Additionally, information on postoperative (PO) progress, more detailed below, was collected during follow-up consultations at the hospital as well as through two phone calls conducted 4 and 6 months after the intervention to assess the presence of lameness and ambulatory function.

### 2.1. Preoperative Management

The preoperative management involved sedating the patient for a radiographic study. The patient was sedated with an intramuscular injection of medetomidine (Medetor 1 mg/mL, Virbac España SA, Esplugues de Llobregat, Spain) at a dose of 100 mcg/kg, methadone (Semfortan, Eurovet Animal Health BV, Handelsweg, The Netherlands) at a dose of 0.4 mg/kg, and midazolam (Midazolam Normon, Laboratorios Normon SA, Tres Cantos, Spain) at a dose of 0.1 mg/kg. Lateral and ventrodorsal pelvic projections were taken, revealing a bilateral APF with a displacement of 6 mm between the fragments in both fracture sites. Post-radiography, the patient was treated with meloxicam (Inflacam 0.5 mg/mL; Virbac España SA, Esplugues de Llobregat, Spain) at a dose of 0.1 mg/kg orally.

After 24 h, the patient was operated on to address both fractures. The premedication protocol was the same as that used for sedation during the previous radiographic study. The patient was induced with propofol (Propofol Lipuro 10 mg/mL; B. Braun, Melsungen AG, Germany) at a dose of 2 mg/kg intravenously and maintained under inhalation anaesthesia using isoflurane (Isoflo; Zoetis Spain SL, Madrid, Spain).

### 2.2. Surgical Technique

The surgical site was prepared by shaving and disinfecting the lumbosacral and gluteal dorsal regions, extending from the midportion of the lumbar spine to below both stifle joints. The patient was positioned in sternal recumbency with a ventral support that kept the hind limbs abducted and the pelvis as horizontally aligned as possible on the surgical table. During the procedure, the main anatomical references of the pelvis were identified: iliac crests, iliac wings, and ischial tuberosities. Safe corridors for the placement of the external fixator (EF) transfixing nails were determined as follows.

- Sacral tuberosity corridor of the ilium, near the origin of the gluteus medius muscle. The pins were inserted at an angle of 10–15° from proximo-medial to disto-lateral to the vertical.
- Corridor of the body of the ilium, in the caudal aspect, along the origin of the gluteus profundus muscle, with pins inserted at an angle of 10–15° to the vertical of the iliac crest.
- Ischial tuberosity corridor, with pins inserted at an entry angle of 10–15° to the vertical.

In total, eight end-threaded, self-tapping 1.2 mm-diameter pins were inserted after performing a predrilled hole: one pin in each iliac crest, another pin in each body of the ilium, and a third and fourth pin in each body of the ischium and ischial tuberosity. Once properly placed by a fluoroscopy-assisted procedure, the pins were used to reduce both APFs, and they were interconnected with a 1.5 mm bar and Meynard clamps (Insorvet, Barcelona, Spain). A pin introduced in the ischium of each hemipelvis was connected directly with a pin inserted in the ilium due to the lack of room for the connection clamp in the main bar.

### 2.3. Postoperative Care

Lateral and ventrodorsal radiographic projections were taken to confirm the fracture reduction. After 24 h, the patient was discharged with a 7-day prescription for cephalexin (Cefaseptin 75 mg; Vetoquinol Especialidades Veterinarias S.A., Alcobendas, Spain) at a dose of 15 mg/kg every 12 h orally and meloxicam (Inflacam 0.5 mg/mL; Virbac España SA, Esplugues de Llobregat, Spain) at a dose of 0.1 mg/kg every 24 h orally.

### 2.4. Follow-Up

Owners were instructed on the daily care of the pin entry sites. Strict confinement of the cat was advised for the first 2 weeks. From the third week until the removal of the fixator, it was recommended to gradually increase the available space for movement, avoiding access to areas where the patient could run or jump. Follow-up radiographs were taken at 21 and 40 days post-surgery. At the 21-day recheck, only a lateral projection was taken in order to review the components of the system. In the last check-up at 40 days, dorsoventral and lateral projections were made. For the dorsoventral projection, the hips were flexed to facilitate better positioning of the patient with the EF. After radiological checking and bone consolidation,, the implants were removed. Subsequently, ventrodorsal and lateral radiographic projections of both hemipelvis were obtained.

In addition to monitoring bone healing, weekly physical examinations were conducted to assess functional and pain assessments and to check the adjustment of the Meynard clamps and pin stability. Two additional rechecks for functionality and pain were performed at 16 and 24 weeks PO.

For the functional assessment, a 6-level scale was established to help determine progress and the final outcome.

- Level 0: no functional or locomotor alterations. The patient walks normally.
- Level 1: mild locomotor alterations, with occasional mild lameness.
- Level 2: mild-to-moderate locomotor alterations, with mild and consistent lameness.
- Level 3: moderate locomotor alterations, with moderate and partially constant lameness.
- Level 4: severe locomotor alterations, with pronounced lameness involving intermittent non-use of the limb.
- Level 5: very severe functional alterations with complete limb disuse.

The pain experienced by the patient during the healing period was assessed using a modified Visual Assessment Scale (VAS) based on the scale described by Flores et al. [29]. This modified scale ranged from A to C, with A indicating very good comfort and C indicating poor comfort for the patient.

- Level A: No apparent signs of pain. The animal exhibits no complaints upon manipulation. Considered normal behaviour.
- Level B: Mild -to-moderate signs of pain. The animal shows some discomfort with occasional spontaneous moans. There may be moderate difficulties in urination and defecation. No other significant behavioural alterations.
- Level C: Severe signs of pain. Inability to handle the patient without accompanying vocalisations. Severe difficulties in urination and defecation. Apathy and depression.

Once radiographic confirmation of the bone consolidation of the fractures was achieved, the removal of the EF was performed. This removal procedure involved sedation and the same protocol described for preoperative management.

## 3. Results

**Figure 1:**
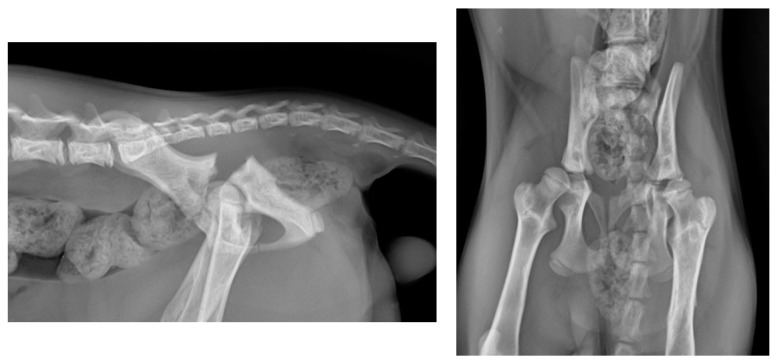
(**Left**): Lateral radiographic projection of the pelvis showing the bilateral APF. (**Right**): Ventrodorsal radiographic projection of the same patient.

The exact cause of the fracture remained undetermined, as the owners did not observe any preceding trauma. No other traumatic injuries were identified upon examination of the patient. The radiographic images revealed the presence of APFs in both hips (Figure 1).

**Figure 2:**
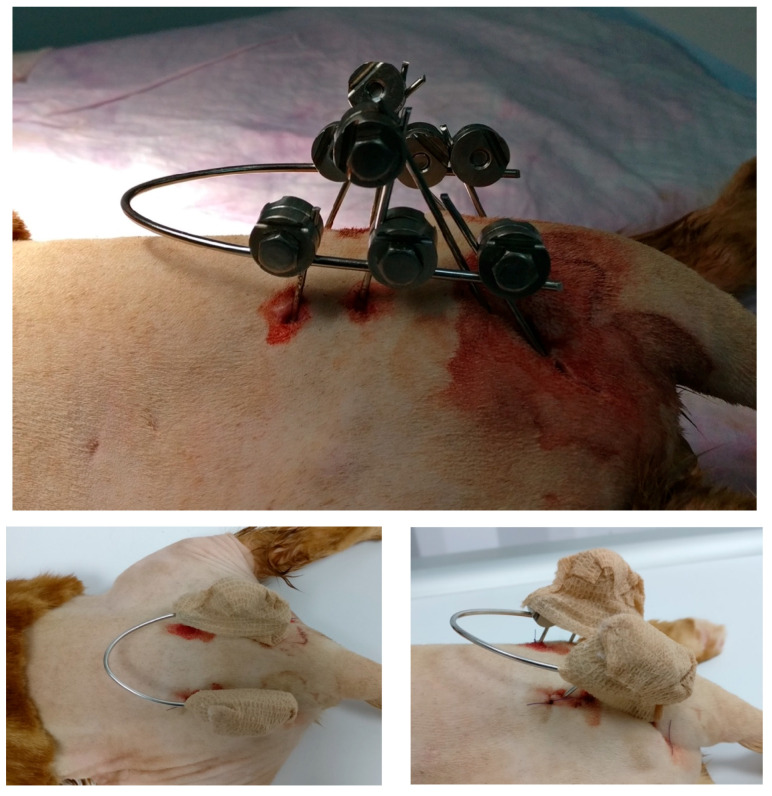
(**Top**): Close-up of the external appearance of the EF. (**Bottom left**): Dorsal view of the EF covered by a protective bandage. (**Bottom right**): Lateral view of the EF.

**Figure 3:**
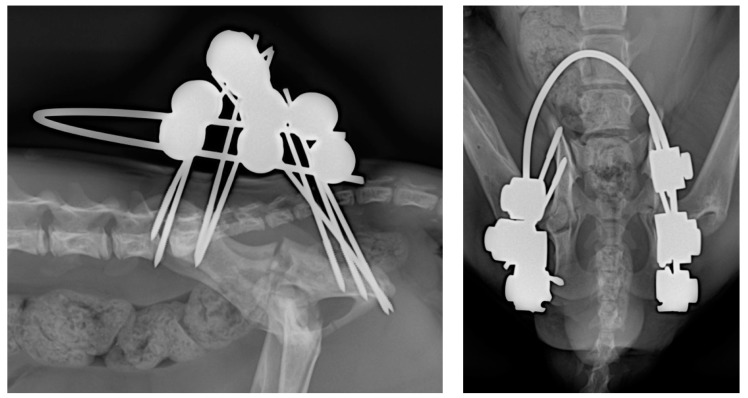
(**Left**): PO lateral radiographic projection. Note the evident reduction in the gap at the fracture site. (**Right**): PO dorsoventral radiographic projection. In the dorsoventral follow-up radiographs, the hips are flexed for better and more comfortable patient positioning.

To stabilise the fractures, an eight-pin fixator with 1.2 mm positive-threaded pins and a connecting 1.5 mm-diameter bar was used (Figure 2 and Figure 3).

**Figure 4:**
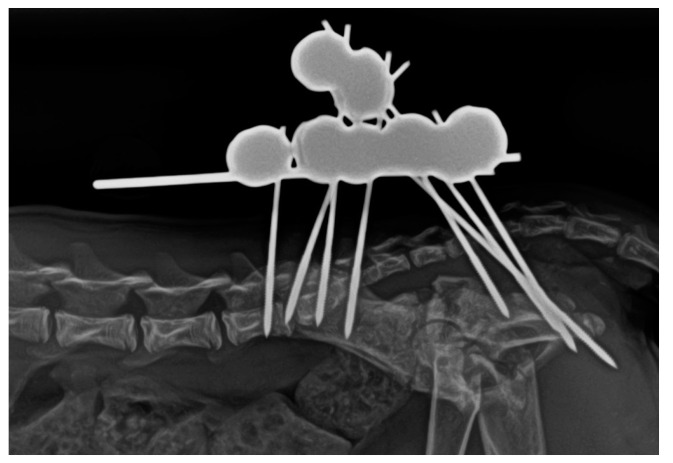
Lateral radiographic projection of the pelvis obtained 21 days after the surgical intervention. Initial radiological signs of healing are observed.

**Figure 5:**
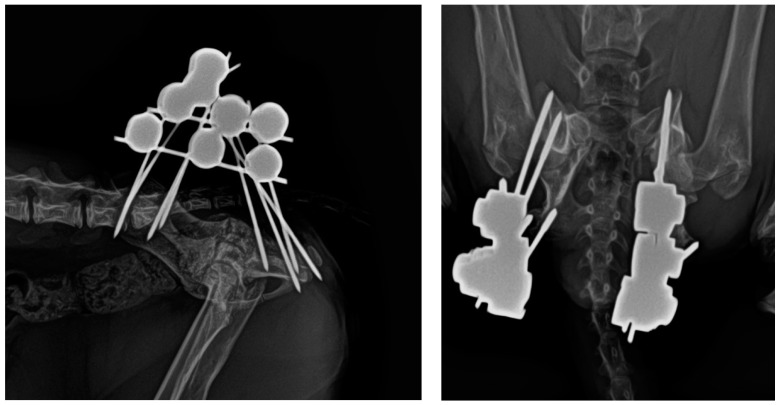
(**Left**): Lateral radiographic projection at 40 days PO. (**Right**): Dorsoventral radiographic projection.

**Figure 6:**
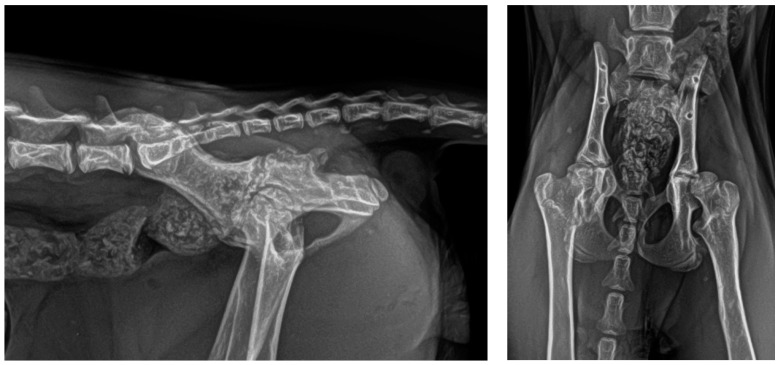
(**Left**): Lateral radiographic projection after the removal of the external fixator. Acceptable healing and proper congruence of both fragments are observed. (**Right**): Dorsoventral radiographic projection. No stenosis of the pelvic canal is evident.

The treatment time with the EF had a total duration of 40 days, with an intermediate radiological check after 21 days (Figure 4) showing initial signs of healing. In the second and final radiological examination at 40 days (Figure 5), complete bone healing was confirmed, allowing for the removal of the system under patient sedation. This procedure was carried out following the aforementioned anaesthetic protocols, and after removal, a new radiological control was performed (Figure 6).

In the initial clinical assessment, the patient had difficulty walking and supporting its own weight with the pelvic limbs, rated at levels 5 and C on both scales. At this point, a neurological examination did not reveal any abnormalities of this nature, although the patient experienced difficulties in urination and defecation, with stranguria and constipation, respectively.

After the surgical intervention, the patient still exhibited a level 5 in functionality and a level C on the pain scale. The use of both pelvic limbs was appropriate, with levels 3 and B during the first 2 weeks of treatment. Some difficulty in extending the spine was observed due to the forward position of the connecting bar between both half-fixators, although this did not prevent the patient from leading a completely normal life with the system. For this reason, it was decided to cut the cranial part of the connecting bar after 21 days of postoperative radiographic control. At the 3- and 4-week controls, the patient showed levels 2 and B on the assessment scales. In weeks 5 and 6, the patient was evaluated at levels 1 and A. After the removal of the system, the patient remained at levels 2 and A for 24 h. Subsequently, the patient returned to complete normality, showing no pain or any alteration in gait and maintaining levels 0 and A in the scheduled follow-ups after 16 and 24 weeks. Information regarding functional and pain evolution during the treatment period is summarised in the following table (Table 1).

Regarding the resistance of the employed system, no loosening of the pins and/or the clamps was recorded due to fatigue in the 40 days of fixation. Throughout the entire healing period, no major complications were experienced, except for the modification of the system at 21 postoperative days and mild, intermittent, and episodic serosanguinous discharge from the pin entry holes observed during the first two weeks of treatment.

## 4. Discussion

In the literature, there are no references to the treatment of spontaneous APFs in cats. Clinical signs compatible with ‘Patellar Fracture and Dental Anomaly Syndrome (PADS)’ were not detected in the patient, as this syndrome is characterised by dental abnormalities, patellar fractures, and other non-traumatic fractures [17,30]. Typically, internal fixation methods dominate the resolution of acetabular fractures [4,12,13,14,15,17]. In human medicine, the use of EF in pelvic fractures at certain stages of treatment has been described [31,32,33,34,35,36] due to its structural advantages [32,33,34,35,36,37,38,39,40], including for acetabular fractures in particular [34,35,36]. EF allows for minimal invasive approaches, resulting in minimal damage to surrounding tissues, with a consequent lower risk of infection, a shorter healing time, and a good level of patient tolerance [25,26,27,28].

In the case presented here, conservative treatment was ruled out due to possible neurological consequences and sustained pain during the healing period [1,2,3,4,5,10]. Additionally, the possibility of osteoarthritis resulting from incomplete fracture site reductions was considered [11,12]. Internal osteosynthesis techniques were excluded due to the small size of the patient, requiring an extensive approach involving small anatomical structures. The possibility of performing a double femoral head and neck excision was considered, but this option was discarded after considering its possible drawbacks like limb shortening, reduced range of motion, muscle atrophy, patellar luxation, and persistent pain and lameness [41].

In this patient, EF was used to reduce both fractures without the need for an open approach, using fluoroscopy for precise manipulation of the bone fragments and achieving good anatomical alignment of the reduction. The use of EF to treat acetabular fractures in cats is uncommon. Only Graville et al. (2018) and Flores et al. (2023) have described this system being successfully applied to acetabular fractures in canines for this type of fracture [23,31]. In other types of pelvic injuries, like sacroiliac luxations, closed reduction has been shown to be effective and provide good results [42], allowing for a faster recovery in the postoperative period. The patient’s size also influenced the choice of the EF system over internal osteosynthesis, as finding suitable implants for a bilateral fracture in a very small cat would have been a challenge [1]. These various factors collectively influenced the decision to employ EF for treating the APFs.

This patient showed moderate to good locomotor function from the early weeks, walking with mild restrictions during the initial two weeks and experiencing minimal limitations throughout the treatment. Severe signs of pain were only detected at the time of presentation and during the first 24 h after surgery. Once the fractures were stabilised with the external fixation and the inflammation decreased, the pain gradually diminished, consequently eliminating the signs of stranguria and constipation induced by it. From the first postoperative week, the patient showed significant improvements, transitioning from levels 5 and C in the assessment scales to levels 3 and B, which is considered a relatively short time for this type of joint fracture. Anti-inflammatory drug treatment was only administered during the first postoperative week, indicating the patient’s good tolerance to the fixation system. The only incident worth noting was related to the positioning of the connecting bar between both sides of the fixator, which was removed after 21 days of treatment. The bar was positioned too far cranially, preventing proper extension of the lumbosacral spine. In the chosen configuration, this bar consisted of a single piece connecting the two parts of the system. Once it was cut, when 50% of bone healing was achieved, both parts of the connecting bar behaved as independent fixators. The patient showed a very good degree of tolerance compared to that observed with open approaches, especially the one described for the acetabular region, which often involves greater trochanter osteotomy or gluteal tenotomy [1,42,43].

Bone healing was recorded after 40 days, and this timeframe is considered acceptable compared to figures obtained in similar cases treated with internal fixation, which often requires up to 60 days [1]. The closed approach employed in this case contributed to a faster recovery, as described in the literature [44]. The use of an EF allowed for easy, quick, and non-invasive implant removal, which is crucial for growing patients. This contrasts with cases where internal fixation is used, which often require a reintervention with an open approach to remove implants, as mentioned in a study by Langley-Hobbs et al. [1]. Additionally, it is highlighted that the diameter of the pelvic canal did not experience any collapse after treatment, which is a common complication and sometimes unavoidable when treating acetabular fractures [1,3,4,5].

During the healing period, no significant complications related to the implant were identified. Only, as mentioned earlier, a modification in the system design had to be made after two weeks of treatment. The system remained intact until the end of the treatment, unaffected by the usual complications of such systems, such as serous discharge from the entry points of the transfixing pins, loosening of the system components, or compromised bone healing [45].

## 5. Conclusions

This work highlights the advantages of EF, demonstrating that its minimally invasive approach and specific characteristics allowed for the successful resolution of a challenging double fracture in a very small and immature patient. The encountered complications were minimal, and the patient showed a good quality of life from the early stages of treatment. Additionally, this type of implant is very cheap, which is useful for its acceptance by the owners.

In summary, pending further research to support these findings, this study suggests that EF should be considered a viable technique for stabilising acetabular fractures in immature cats.

## Figures and Tables

**Table 1:** Data obtained in different follow-up controls assessing the patient’s functional status and pain according to the described assessment scales. PrO: preoperative; PO: postoperative; W: week; EXP: implant removal.

	PrO	PO	W1	W2	W3	W4	W5	W6	EXP	W16	W24
Functional Scale	5	5	3	3	2	2	1	1	2	0	0
Pain Scale	C	C	B	B	B	B	A	A	A	A	A

## Data Availability

Data are contained within the article.
